# Medical, Geographical, and Agricultural Report of a Committee Appointed by the Madras Government, to Inquire into the Causes of the Epidemic Fever Which Prevailed in the Provinces of Coimbatore, Madura, Dindigul, and Tinnivelly, during the Years 1809, 10, 11

**Published:** 1817-05

**Authors:** 


*Medical, Geographical, and Agricultural Report of a Committee appointed by the Madras Government, to inquire into the Causes of the* Epidemic Fever *which prevailed in the Provinces of Coimbatore, Madura, Dindigul, and Tinnivelly, during the Years* 1809, 10, 11. President, Dr. W. Ainslie; 2d Member, Mr. A. Smith; 3d Member, Dr. M. Christie. Octavo, pp. 270, London, 1816.

From the Medico-Statistico-Agricultural nature of this volume, it can only be interesting, *as a whole,* to those who visit the Eastern world; and consequently, it will have no general circulation through the profession. To prevent the purely *medical* part from being lost to the Faculty, we propose transfusing it into the pages of our Journal, in our usual terse analytical manner. An epidemic, spreading its ravages from Cape Comorin to the Banks of the Cavery—from the Ghauts to the coast of Coromandel, and sweeping to the grave 106,789 persons, presented a noble field for investigation—an unbounded theatre for the acquisition of medical knowledge! But, alas! the richness of the soil seems only to have rendered indolent the cultivator; and a miserable stunted harvest has been gathered from the most luxuriant plains and vallies on which the sun of science ever shone! There appears to be a mephitic vapour hanging over the medical atmosphere of our Asiatic possessions, which paralyses the mental faculties, and withers every shoot of professional ardour, emulation, and enthusiasm! We bewail the spell that enthrals the minds of our medical brethren in the East, and we despair of breaking it. The glimmering taper which we are now contemplating, is badly calculated to dispel the Cimmerian darkness which still hangs over the nature and propagation of epidemic visitations; but we must be content to make the most of the scanty materials which are here collected.

The sections of this work which we propose to analyse, are the 4th, 5th, and 6th, treating of the causes, nature, and treatment of the epidemic.

1. *Causes.* Since the time of Hippocrates, *atmospheric vicissitudes* have been deemed insalutary; and Hoffman set them down as the general remote cause of epidemic fever. The committee believe that Sydenham's *"Secret Constitution of the Air"* is as good an explanation as can be given. We believe not; but shall not stop here to discuss the point. They justly, however, remark, that an erroneous opinion has prevailed, that marsh miasmata can only be engendered in low swampy situations, "though it is well known that noxious vapours from woods, especially if thick and ill ventilated, are as certainly a source of the same mischief." This second source was very abundant in several of the ravaged provinces, many parts being so covered with wood, jungle, and rank vegetation, as to be nearly impervious. Another supposed origin of febrific miasmata was in the salt marshes found in the Tinnevelly and Ramnad districts, where the fever raged with uncommon severity. The committee are of opinion, that marshy situations are not sufficient to render fevers epidemic; there is required the super-agency of a close, moist, and sultry heat, with imperfect ventilation. Such an offensive condition of the atmosphere was but too often experienced in several of the low tracts of these districts during the sickly season, and was pregnant with the most baleful consequences. Although great deviations from the natural order of climate are, fortunately, not very frequent in these regions, yet, as in the present instance, they do sometimes take place; and are always followed by disastrous results. Major Orme informs us, that in the month of March, the S. W. monsoon broke completely over the western Ghauts, and descended in vast floods over the Coromandel side of the Peninsula, destroying crops just ready to be cut, sweeping away many of the inhabitants, and ultimately, by creating a powerful evaporation during a sultry heat, producing an epidemic disease very fatal in its consequences.

The effects of those miasmata engendered amongst woods and jungles have been too well authenticated to require additional testimony. As electricity has been said to promote putrefaction in animal bodies, the committee query how far this fluid, which was very abundant in the atmosphere during the sickly seasons, may not have assisted in producing a distempered state of the air. We think this is a very questionable cause of epidemia.

The predisposing causes of remittent and intermittent fevers are well known to be those which operate by producing debility, as bad diet, fatigue, exposure to cold and damp, grief, mental anxiety, &c. This is illustrated by a remarkable exemption from disease, among the troops stationed at Madura, while the poor inhabitants of the garrison were swept off by sickness. The same was obserevd at Dindigul, where two deaths only occurred among three companies of troops, while the needy inhabitants of the town were dying by hundreds.

Of the *exciting* causes, the committee considered exposure to cold and damp, while the body had been relaxed by preceding heat, and the solar influence, as the most powerful.

"The heat of the early part of the nights induced many of the natives to sleep in the open air, by which means they became exposed, while yet perspiring, to the chill fogs and damps of the morning." P. 116.

2. *Nature and Types of the Epidemic.* This fatal fever did not differ essentially from the common endemic of the country. Its epidemic tendency, on the present occasion, was altogether ascribable to the *causes* enumerated in the preceding section. It is either remittent or intermittent, according to the constitution, treatment, and season of the year. People by nature delicate and irritable, or rendered so by irregularities, or want of care, are sometimes attacked by the disease in the remittent form, proving bilious or nervous, as the constitution inclines. The same happens to the more robust, when improperly treated, as where bark is given early, and before proper evacuations have been premised. As the season becomes hotter, too, the remitting form prevails over the intermittent. Males suffered more than females, and young people and those of middle-age more than old people and children. The remittent form sometimes makes its approaches very insidiously. The patient feels himself out of sorts for a few days; his appetite fails him; he has squeamishness, especially at the sight of animal food; universal lassitude; alternate heats and chills; stupid heaviness, if not pain in the head. The eyes are clouded; the ears ring; the bowels are invariably costive. In other cases, the enemy approaches rapidly; and rigors, great prostration of strength, vertigo, nausea, or vomiting, usher in the disease. The first paroxysm, which is often attended with delirium and epistaxis, after continuing an indefinite period, with varying symptoms, terminates in a sweat; not profuse and fluent, as after a regular hot fit of ague, but clammy and partial, with the effect, however, of lowering the pulse and cooling the body, but not to the natural standard. The latter still feels dry and uncomfortable; the pulse continuing smaller and quicker than it ought. This remission will not be of long standing, without proper remedial measures. A more severe paroxysm soon ensues, ushered in by vomiting (sometimes of bile), and quickly followed by excessive heat; delirium; great thirst; difficult respiration; febrile anxiety; parched and brownish tongue. The next remission (if it do take place) is less perfect than the first, and brings still less relief. In this way, if medicine, or a spontaneous purging do not check the disease, it will run its fatal course, each succeeding attack proving worse than its predecessor, till exhausted nature begins to give way. The pulse declines; the countenance shrinks, and looks sallow; the eyes become dim; "*the abdomen swells from visceral congestion;*" the stomach loathes all food, when hiccup, stupor, and low delirium usher in death. Such severe cases, the committee think, were, in general, owing to neglect or blunders at the beginning of the disease.

Intermittents were more intractable, as well as more common. The epidemic was void of any contagious character, except in cases that were allowed to run into the low continued form; and even here, the contagion was circumscribed within very narrow limits. The types were, the simple tertian, the double tertian, the quotidian, the quartan, and the irregular. The following will give some idea of the relative numbers of these forms.—A native detachment at Dindigul, 255 strong, suffered in the following proportion: Simp. tert. 30; doub. tert. 26; irreg 24; quotid. 13; quart. 4. The quotidian form was well marked, returning at nearly equal periods, often attacking weak constitutions, and leaving but little time for taking the bark. It was more apt to occasion visceral obstructions and œdematous swellings than any other form of the disease. The quartan was rare, but obstinate, and frequently productive of splenic obstruction and dropsy. The irregular were very troublesome, and seemed to correspond with Hoffman's semi-tertian.

The Tamool, or native practitioners, ascribe the epidemic fever chiefly to two causes — a superabundance of moisture in the air and earth, and the bad quality of the water, owing to unwholesome solutions. We think there is much truth in their opinions, and have had reason to think ourselves, that the water, as well as the air, becomes impregnated with morbific miasmata.

*Treatment.* On the first appearance of the epidemic, no time was lost in clearing out the bowels by brisk purgatives; and soon after the medicine had ceased to operate, the cinchona was prescribed, observing this rule respecting it, that, the nearer the time of giving the last dose of bark for the day is brought to the period of attack of the cold stage, the more likely will it be to accomplish the purpose intended. From six to eight drachms of the fresh powdered bark, taken in substance, was commonly sufficient to keep off a fit, especially if given in the four or five hours preceding the paroxysm. Some of the native stomachs could not bear the powder, unless mixed with ginger, or given in infusion or decoction, with tinct. cinchonæ and conf. aromat. As the bark sometimes constipated, a few grains of rhubarb were added, or laxative glysters used. Thirty or forty drops of laudanum, with half an ounce of the acetate of ammonia, given at the commencement of the hot fit, often had the effect of shortening it, sustaining the strength, and rendering the stomach retentive. When the perspiration begins to flow, the drink ought to be tepid; but when the body is hot and the skin dry, cold water is both grateful and salutary. The bark must be continued for some time after the fever disappears, to prevent recurrence. The committee, as was to be expected, from the schools of debility and putrescency in which they were educated, and from the known and rooted prejudices of these scholars, declaim against purgatives in this fever, lest they be productive of mischief, by occasioning irritation, *debility*, and ultimately an obstinate disease—*mindful of the lesson that was taught them in early life*, by the writings of the judicious Hoffman," &c. &c. We quote this passage, not to say that we think drastic purgatives necessary in the simple form of intermittent, for we know that they are *unnecessary*, and sometimes hurtful; but to shew that the committee were genuine disciples of Hoffman and of Spasm; and consequently, that we are not to look for any thing beyond the spell-bound circle of those fallacious theories!

When the fever, as was too often the case, ran its course some days unchecked by medicine, then the case was altered, for abdominal congestion and visceral obstruction soon took place, and a dangerous state of the disease was induced. In these distressing circumstances, change of climate was necessary, and a course of calomel. When the mouth became affected, some of the most unpleasant symptoms disappeared, and then the bark was administered with more safety.

"There are a description of medical men in this country [India], say the committee, who suppose that, in hot climates, bark given for intermittent fevers has the effect of bringing on abdominal obstructions, if calomel is not at the same time administered; but to this opinion we cannot subscribe."

Here the old changes are rung, and the superannuated dreams related of the dreadful effects of calomel!

"*We* see no good reason why this *acrid mineral* should be given, however necessary it may be to alter the habit in more serious attacks: it is an *irritating* and *debilitating* medicine; it is very apt to *sicken the stomach,* and produce dyspepsia; and *must therefore* prove particularly objectionable at all times in delicate habits; &c."

How will the anti-mercurial drivellers of this country exult, when they hear such language from men high in office in our Indian possessions! But we have long ceased to couple abilities with rank, or true knowledge with length of years. We every day see error riveted by time, prejudice confirmed by experience. The poet indeed avers, that—

> "The Soul's dark cottage, batter'd and decay'd,
> Admits new light through chinks which time has made."

But this is far from being *always* the case in our "conjectural art;" on the contrary, the glimmerings of reason, and the light of patient pathological investigation, are too often excluded by that cold-blooded obstinacy, and adamantine prejudice, which we occasionally see encircle the hoary head of experience! It is pleasing, however, to remark examples of a contrary nature. Dr. Balfour, who held a still higher office than the committee, declares as follows:

"In the space of *twenty years* I cannot say that I met with *any case,* in which I conceived bark to be properly administered, and in sufficient quantity, where it *ever failed* of securing the patient in the end." *Sol-Lunar Influence,* 1790.

Yet *fifteen years* corrected the experience of *twenty;* and in 1805 he comes to the following conclusions:

"Considering that obstructions of the liver very frequently shew themselves in the common fevers of this country, and may, with great reason, be suspected, in a certain degree, *in all,* we cannot hesitate to admit, as an *essential* and valuable principle, in the cure of fevers, *the introduction of mercury into the system, so as to affect the mouth in a moderate degree,* with the view of removing obstructions, or other morbid affections of the liver; of obtaining natural secretions, and of its thus contributing, *with the other means* that have been described, to a speedy and permanent cure." *Preface to Collect. of Treatises, &c.* 1805.

Thus we see that twenty years' experience enabled Dr. B. to lay down a plan of cure, which never, in *any case,* failed during that immense period; and yet fifteen years afterwards a most important alteration is made, and a new principle of cure is admitted! These contrasting passages will lessen, in some degree, the cry of exultation which the committee may inspire among the debility and putrescency party.

The committee not unfrequently met with obstinate intermittents, unaccompanied apparently by visceral obstruction, in which bark was unavailing. They sometimes tried with success sulphuric æther in doses of ʒjß, taken at the approach of the cold fit; and also full doses of laudanum. The sulphate of zinc did not answer. The Hindoo practitioners have used arsenic in intermittent fevers time immemorial, and entertain a high opinion of its virtues; but the committee do not approve of it much, though it sometimes succeeded when all other remedies had failed. The cold affusion was useful in the hot fits; nay, daily immersion in the sea sometimes proved the happy means of checking agues which had baffled every other exertion. A blister to the nape of the neck will sometimes check the recurrence of the cold fit. A full dose of the *tinct. rhei et aloes*, at bed-time, was found by Mr. Tait, of Trichinopoly, to stop agues that resisted every other remedy. Notwithstanding all our endeavours, the disease will sometimes run on to coma and death.

"In such cases, calomel or the blue pill, continued till the mouth is a little affected, *even when no obstruction has taken place*, is *often* found to be of the greatest service." 145.

On this we shall make no comment; the fact speaks for itself. Alarming bowel complaints sometimes supervene on long-protracted intermittents; not attended with much straining, but of an obstinate and debilitating nature, requiring opiates, weak cretaceous mixtures, and aromatics. They too often proved fatal, especially among the natives.

Œdematous swellings and ascites not unfrequently supervene from pure debility. These, where no visceral obstruction prevailed, were best treated by tincture of squills, ginger, and tinct. cinchonæ, together with frequent friction with dry flannel, and proper attention to the ingesta. But when the bowels were firm, and there was any suspicions of organic derangement in the abdomen, calomel in small doses was conjoined with the squills; or what answered better, the pilula hydrargyri.

This fever coming on patients who had previously suffered from liver affections or dysentery, assumed an alarming and complex form, requiring the nicest management. Bark was here to be used with great caution. Even the infusion and decoction were dangerous, where there was any pain or uneasiness in the right side. A blister, without loss of time, was then applied, and mercury had recourse to.—℞. Pil. hydrargyri gr. vj; pulv. ipecac. gr. iij; opii gr. ß; fiant pilulæ tres. Sumatur una ter die; resuming the use of the cinchona as the hepatic symptoms subside. Sometimes the two remedies were combined, where the hepatic affection was chronic and not very obtrusive. An issue in the right side, with bitters and tonics, often proved serviceable. Change of air was superior to all other means, and diet of course required constant attention. Gentle exercise; flannel next the skin, especially where hepatic affections existed; and the most scrupulous attention to the state of the bowels.

When, from the appearance of the symptoms, a fever of the remittent kind is approaching, emetics are improper; in this case, the committee recommended six grains of calomel and six of James's powder, to be taken in the course of twelve hours, which will generally produce copious evacuations, and sometimes diaphoresis.

"On the second day, when the paroxysm will, in many cases, be found every way more severe than on the first, no time is to be lost in having recourse to mercury; *the remedy which, at such times, can best be relied on for producing a proper intermission.* Seven or eight grains of calomel, with three grains of camphor, are to be well rubbed together, and made into four pills, one of which is to be taken every three hours during the day. These will often have the desired effect, if continued for two or three days, by producing a desirable change in the habit, and so favourable a remission, that the bark may be given with safety." 154.

If this be not a decisive evidence in favour of the *anti-febrile* powers of mercury on the constitution, we know not what evidence would carry conviction to the minds of the declaimers against the medicine. It is the more satisfactory, as it comes from the anti-mercurial party themselves, surrounded with the prejudices of debility and putrescency.

The committee cannot speak decidedly respecting sol-lunar influence on this fever, and on relapses; but they appear to deny the inferences which Dr. Balfour and others have drawn. The principal native remedies employed by the Tamool practitioners were, white arsenic, about the 15th part of a grain, twice a day; the barks of the Swietenia febrifuga, and melia Azadirachta; the Cutcaranja nut; the Chukkoo (Amom. Zingib.); the Sison Ammi; bark of the Acacia Arabica, and Tellicherry bark.

Scanty as are the materials collected in this Report, we have thought them worthy of a place in a Journal professedly aiming at being the depositary of the medical information of the day. The steady light of pathological researches beamed not on the path of the committee; while the *ignes fatui* of the schools have occasionally led them astray. But while we deplore the want of that enthusiasm which, from such an ample field for observation, would have drawn copious stores of invaluable knowledge, we must still allow, that the report of this committee contains much important matter, that may prove food for useful reflection.

We have lately heard it urged, that the causes of intermittent and remittent fevers must necessarily be sought in low and marshy situations; whereas the testimony of unquestionable writers, and this document particularly, proves, that febrific miasmata may rise, under certain conditions, from almost any soil; and what is still more extraordinary, that these febrific miasmata may be carried, by currents of air, to a distance far exceeding what has been laid down by some most respectable writers on the subject. This epidemic of India spread its poisonous breath from south to north, in the direction of the monsoon, and was confidently believed by the natives to have its sources in the Pylney mountains, whose overgrown woods, unventilated vallies, and stagnant marshes, could not fail to engender a more rapidly dangerous condition of the atmosphere, than that brought about by the same general causes on the drier and less woody plains of the eastern ranges of the peninsula.

The observations of the committee are corroborated by the testimony of others, particularly Zimmerman and Jackson.

"Fevers of this sort (says the latter) arise in particular countries, or districts of a country. They travel in certain tracts: sometimes confined to narrow bounds; at other times they are more widely diffused." *Medical Dep. Brit. Army, p.* 212. *See also Zimmerman's* "*Experience,*" *vol. ii. p.* 155.

In this dreadful epidemic, too, as no person found it convenient to write a book on its contagious properties, no infection was traced, and no paper war was the consequence.

It is greatly to be lamented, that some of the *energetic* modes of treatment lately introduced into the *methodus*

*medendi* of fever had not been tried in the remittent forms of the eastern epidemic. It does not appear that a lancet was wet in any part of the epidemic range from Cape Comorin to the Cavery; and therefore it is in vain for our Oriental brethren to say that it would not have been useful, when they never gave it a trial. The evidence, however, in favour of MERCURY is most unequivocal, and will probably silence, if any thing can, the clamour which has been raised against it in this country, by a party by no means distinguished for genius, erudition, or solid talents.

